# Efficacy analysis of remifentanil mild sedation anesthesia for painless gastroscopy

**DOI:** 10.3389/fphar.2025.1692910

**Published:** 2025-11-06

**Authors:** Dongfeng Xi, Huiling Zhang, Mengyuan Gu, Yao Wang, Jiangbo Qu, Huibin Mao, Lina Mi, Bin Li

**Affiliations:** 1 Department of Anesthesiology, Yangquan Coal Industry Group Co., Ltd. General Hospital, Yangquan City, Shanxi, China; 2 Endoscope Room, Yangquan Coal Industry Group Co., Ltd. General Hospital, Yangquan City, Shanxi, China

**Keywords:** remifentanil, sedation, painless gastroscopy, propofol, complications, anesthesia recovery

## Abstract

**Purpose:**

To investigate the clinical efficacy of remifentanil mild sedation versus propofol deep sedation for anesthesia during painless gastroscopy.

**Methods:**

A total of 980 patients undergoing painless gastroscopy at our hospital’s endoscopy center from January to May 2025 were enrolled and randomly divided into a control group (490 cases, propofol-etomidate mixture intravenous injection) and an observation group (490 cases, remifentanil intravenous injection) using a computer-generated random sequence with sealed envelope allocation. Intraoperative vital sign changes, complication rates, time to ambulation, anesthetic dosage, and patient satisfaction were compared between the two groups.

**Results:**

The overall complication rate in the observation group was 0.6%, significantly lower than 64% in the control group (P < 0.05). 76.3% of patients in the observation group experienced intraoperative blood pressure and heart rate fluctuations, compared to 78.6% in the control group, with no significant difference (P > 0.05). The observation group demonstrated significantly shorter ambulation time (17 ± 1.8 vs. 25 ± 3.6 min, P < 0.01) and higher satisfaction rates (patients: 92% vs. 94.4%; clinicians: 98% vs. 95%, P < 0.05) than the control group. Multivariate logistic regression analysis showed that remifentanil use was an independent factor for reducing complications (OR = 0.12, 95%CI:0.05–0.28, P < 0.001).

**Conclusion:**

Remifentanil intravenous injection combined with lidocaine gel for mild sedation anesthesia effectively alleviates discomfort during gastroscopy. Compared with propofol-based deep sedation, it demonstrates a lower complication rate and higher safety.

## 1 Introduction

With the aging population and increasing health awareness, demand for painless gastroscopy is rising, posing greater challenges to anesthesiologists managing patients with multi-organ dysfunction or difficult airways. Traditional anesthesia for painless gastroscopy often involves deep sedation with propofol combined with low-dose opioids, which may lead to complications such as glossoptosis, airway spasm, and injection pain. To explore safer and more effective anesthesia regimens, some scholars have proposed moderate or mild sedation using different drug combinations (e.g., midazolam with sufentanil) (Hamed et al., 2019). However, the optimal depth of sedation remains controversial. This study evaluates the feasibility, safety, usage characteristics, and clinical efficacy of remifentanil intravenous injection combined with lidocaine gel surface anesthesia for painless gastroscopy.

## 2 Materials and methods

### 2.1 General information

Approved by the hospital’s ethics committee of the Yangquan Coal Industry Group Co., Ltd. General Hospital (Approval No.: YMZYLLM2024030; Ethical approval date: 6th., Sept., 2024) and with informed consent, 980 patients undergoing painless gastroscopy at the Endoscopy Center of Yangquan Coal Industry Group General Hospital between January and May 2025 were enrolled. Exclusion criteria included allergies to study drugs, severe cognitive impairment, and severe cardiopulmonary decompensation. Patients were classified as American Society of Anesthesiologists (ASA) I–III, aged 18–86 years, with 508 males and 472 females, and a body mass index (BMI) of 16–33 kg/m^2^. They were randomly divided into control and observation groups (490 cases each) using a computer-generated random sequence. Group allocation was concealed via sealed envelopes, and no missing data were observed in this study. The two groups were comparable in gender, ASA classification, age, BMI, proportion of patients with difficult airways, examination duration, and proportions of biopsy and polypectomy cases (P > 0.05; Table 1).

**TABLE 1:** Comparison of general information between the two groups of patients.

Indicator	Control group (n = 490)	Observation group (n = 490)	*t/χ* ^*2*^	*P*
Age (years)	56.6 ± 5.3	55.9 ± 7.1	1.749	0.081
Gender Composition (Male/Female, cases)	261/229	247/243	0.802	0.37
Body Mass Index (kg/m^2^)	21.6 ± 4.7	22.2 ± 6.5	−1.656	0.098
ASA Classification (Ⅰ/Ⅱ/Ⅲ, cases)	364/105/21	379/83/28	3.876	0.144
Combined Difficult Airway Factors (cases, %)	138 (28.2%)	147 (30%)	0.401	0.527
- Snoring (cases)	57	69	1.311	0.252
- Mallampati Grade Ⅲ/Ⅳ (cases)	45/11	54/8	0.910	0.340
- Short Neck (Thyromental Distance <6 cm) or Obesity (BMI≥30) (cases)	25	16	22.062	0.151
Metabolic Equivalent (MET)	6.5 ± 1.8	6.3 ± 2.3	1.516	0.130
Medical history				
- No Systemic Diseases (cases, %)	256 (52.2%)	275 (56.1%)	1.484	0.223
−1 Systemic Disease (cases, %)	101 (20.6%)	94 (19.2%)	0.314	0.575
−2 Systemic Diseases (cases, %)	87 (17.8)	71 (14.5%)	1.932	0.165
- ≥3 Systemic Diseases (cases, %)	46 (9.4%)	50 (10.2%)	0.184	0.668
- Hypertension (cases)	97	106	0.502	0.478
- Diabetes (cases)	76	63	1.416	0.234
- Coronary Syndrome (cases)	75	60	1.932	0.165
- Cerebral Infarction (cases)	67	79	1.158	0.282
- Renal Failure (cases)	23	18	0.636	0.425
- Others (Digestive System Diseases, etc.)	77	61	2.160	0.142
Examination Duration (min)	10.6 ± 3.5	11.1 ± 4.8	−1.864	0.062
Biopsy during Gastroscopy (cases)	121	128	0.264	0.608
Polyp Removal during Gastroscopy (cases)	15	22	1.376	0.241
Biopsy + Polyp Removal (cases, %)	136 (27.8%)	150 (30.6%)	0.968	0.325

### 2.2 Methods

Preoperative outpatient examination data included blood biochemistry, complete blood count, and electrocardiogram (ECG). Anesthesia assessment was performed 1 day prior to the procedure, and informed consent for anesthesia was obtained. On the day of the examination, after establishing intravenous access, patients entered the procedure room where continuous monitoring of ECG, non-invasive blood pressure, and pulse oximetry was initiated. All patients were instructed to gargle with 10 g of 2% lidocaine hydrochloride mucilage (Ze Heng, Sichuan Jianneng Pharmaceutical Co., Ltd., batch number 4240302) prior to the procedure. After positioning, patients received oxygen via a facial mask at a rate of 4–6 L/min.

In the control group, sufentanil injection (Renfu Pharmaceutical Group Co., Ltd., batch number AB40400511) was administered intravenously at a dose of 0.05 μg/kg, followed by a combination of propofol (Disining, Guangdong Jiabo Pharmaceutical Co., Ltd., batch number 7A240501) and etomidate (Fuerli, Jiangsu Nhwa Pharmaceutical Co., Ltd., batch number TYT24G39) (150 mg:10 mg) at a dose of 0.2–0.3 mL/kg (equivalent to 1.2–1.8 mg/kg propofol +0.08–0.12 mg/kg etomidate). After the eyelash reflex had disappeared, an additional 2–3 mL of the propofol-etomidate mixture was administered intravenously to facilitate endoscope insertion. During the gastroscopy, intermittent intravenous injections of 2–3 mL of the propofol-etomidate mixture were administered to maintain the depth of sedation (Ramsay Sedation Score 5–6 points). Vasopressors were used to maintain optimal hemodynamic status based on changes in vital signs, and measures such as chin lift, nasopharyngeal airway insertion, and mask ventilation were employed to maintain airway patency depending on real-time respiratory status.

In the observation group, remifentanil (Ruijie, Renfu Pharmaceutical Group Co., Ltd., batch number AC4050461, 1 mg/250 mL normal saline) was administered intravenously at a dose of 0.8–1.2 μg/kg. After the patient reached Ramsay Sedation Score 2–3 points (mild sedation), an additional 2–3 mL of remifentanil (1 mg/250 mL, equivalent to 0.8–1.2 μg/kg) was administered intravenously to facilitate endoscope insertion. Depending on the procedure duration or specific steps (e.g., biopsy or polypectomy), 1.5–2.5 mL of remifentanil was intermittently administered to maintain mild sedation. Vasopressors or corresponding measures were used to maintain stable vital signs. Sedation depth was assessed using the Ramsay Sedation Scale (Table 2).

**TABLE 2:** Criteria for assessing the depth of sedation.

Assessment item	Mild sedation	Moderate sedation	Deep sedation	General anesthesia
Ramsay Sedation Score	2–3 points	4 points	5–6 points	-
Response	Normal response to verbal stimuli	Purposeful response to verbal or tactile stimuli	No response to non-noxious stimuli, but response to noxious stimuli	No response to noxious stimuli
Ventilation Function	No impact	Adequate, no intervention required	May be inadequate, intervention may be required	Often inadequate, intervention often required
Cardiovascular Function	No impact	Usually maintained	Usually maintained	May be impaired

### 2.3 Observation indices

Changes in intraoperative vital signs were recorded for both groups at the following time points: after positioning (T1), after anesthesia induction (T2), during endoscope insertion (T3), and every 3 min thereafter until T4, T5, and T6. All vital sign data were presented as mean ± standard deviation (Table 3).

**TABLE 3:** Intraoperative vital sign changes (mean ± SD) between two groups.

Time point	Group	Systolic blood pressure (mmHg)	Diastolic blood pressure (mmHg)	Heart rate (bpm)
T1	Control	132.5 ± 11.3	82.4 ± 7.6	78.5 ± 9.2
	Observation	130.8 ± 10.5	80.6 ± 8.1	76.9 ± 8.7
T2	Control	108.5 ± 9.6	65.3 ± 6.2	62.1 ± 7.5
	Observation	125.3 ± 10.2	75.2 ± 7.3	72.3 ± 8.1

The incidence of intraoperative complications (including airway obstruction, body movement requiring restraint, agitation, choking, SpO_2_<90%) and postoperative complications (nausea, vomiting) were compared between the two groups (Table 4). Hiccups and anterograde amnesia were not classified as complications due to their lack of clinical relevance.

**TABLE 4:** Comparison of complication rates between two groups (n, %).

Complication type	Control group (n = 490)	Observation group (n = 490)	*χ* ^*2*^	P
Intraoperative body movement	244 (49.8)	0 (0)	321.56	<0.001
SpO_2_<90%	127 (26.0)	2 (0.4)	148.32	<0.001
Postoperative nausea	3 (0.6)	3 (0.6)	0.00	1.00
Total complications	312 (63.7)	5 (1.0)	428.71	<0.001

Additionally, the time to ambulation, dosage of anesthetic drugs, patient satisfaction, and endoscopist satisfaction were compared. Satisfaction was assessed using a validated hospital-specific questionnaire (Cronbach’s α = 0.86), with “satisfied” defined as a score ≥8, “neutral” as 4–7, and “dissatisfied” as ≤3. Satisfaction rate was calculated as [number of satisfied subjects]/490% × 100%.

### 2.4 Statistical analysis

SPSS 24.0 software was used for statistical analysis. Measurement data were expressed as mean ± standard deviation, and comparisons between groups were performed using independent sample t-tests. Counting data and rate comparisons were analyzed using the χ^2^ test. Bonferroni correction was applied for multiple comparisons, with P < 0.008 considered statistically significant. Multivariate logistic regression analysis was performed to identify independent factors associated with complications (variables included age, ASA classification, and anesthetic regimen). Effect sizes were reported as odds ratios (OR) or mean differences with 95% confidence intervals (CI). A P-value <0.05 was considered statistically significant before correction.

## 3 Results

### 3.1 Intraoperative vital sign changes

In the control group, 385 patients (78.6%) experienced a decrease in blood pressure and heart rate after the start of gastroscopy, with 86 patients requiring vasopressors. The remaining 105 patients (21.4%) had stable or slightly elevated heart rates and blood pressures compared to preoperative levels. In the observation group, 374 patients (76.3%) showed an increase in blood pressure and heart rate during gastroscopy, with 22 patients requiring nitroglycerin or nicardipine to lower blood pressure. The remaining 116 patients had stable or slightly decreased heart rates and blood pressures compared to preoperative levels. There was no statistically significant difference between blood pressure and heart rate fluctuations between the two groups (P > 0.05).

### 3.2 Complication rates

During gastroscopy, 244 patients (49.8%) in the control group experienced body movement requiring passive restraint, 127 patients (26.0%) had decreased pulse oximetry or glossoptosis (lowest value 83%), requiring chin lift (101 cases), nasopharyngeal airway insertion (12 cases), mask ventilation after interrupting the procedure (11 cases), airway spasm, and intravenous dexamethasone administration (3 cases). Additionally, 85 patients (17.3%) experienced hiccups, 9 patients (1.8%) had injection pain, and 12 patients (2.4%) had muscle twitching. The amnesia rate for gastroscopy was 100%, with 3 patients (0.6%) experiencing postoperative nausea but no vomiting. The overall complication rate was 64.0%.

In the observation group, 2 patients (0.4%) experienced decreased pulse oximetry (lowest value 89%), which improved after deep breathing instructions. No other intraoperative complications occurred. Five patients (1.0%) experienced postoperative nausea, and 15 patients (3.1%) required one swallowing action when the endoscope reached the esophageal opening for smooth passage (not classified as a complication). The overall complication rate was 0.6%. There was a statistically significant difference between the two groups (P < 0.05, OR = 0.01, 95%CI:0.003–0.03).

Multivariate logistic regression analysis showed that remifentanil use was an independent factor for reducing complications (OR = 0.12, 95%CI:0.05–0.28, P < 0.001), after adjusting for age and ASA classification.

### 3.3 Time to ambulation, anesthetic drug dosage, and satisfaction rates

The time to ambulation in the control group ranged from 19 to 35 min, with an average of (25 ± 3.6) minutes, while in the observation group, it ranged from 15 to 22 min, with an average of (17 ± 1.8) minutes. There was a statistically significant difference between the two groups (P < 0.01, mean difference = −8.0 min, 95%CI: 9.2 to −6.8).

The dosage of propofol-etomidate mixture used in the control group was (18.3 ± 2.2) mL, while the dosage of remifentanil used in the observation group was (22.5 ± 3.8) mL.

Patient satisfaction in the control group was 94.4%, and endoscopist satisfaction was 95.0%. In the observation group, patient satisfaction was 92.0%, and endoscopist satisfaction was 98.0%. There was a statistically significant difference in endoscopist satisfaction between the two groups (P < 0.05, OR = 1.52, 95%CI:1.03–2.25), while no significant difference was observed in patient satisfaction (P > 0.05, OR = 0.78, 95%CI:0.52–1.17).

## 4 Discussion

Anesthesia for painless gastroscopy and colonoscopy can eliminate the mechanical stimulation and discomfort caused by gastrointestinal endoscopy diagnosis and treatment, serving as an important component of comfortable diagnosis and treatment. However, both doctors and patients bear the anesthesia risks, especially for high-risk patients such as the elderly or those with difficult airways, a history of gastrointestinal surgery, and other factors, where the incidence of complications significantly increases (Ribeiro Gomes et al., 2020; Hamre et al., 2020). To reduce anesthesia risks, some scholars have adopted strategies such as reducing the dosage of propofol and using other sedative drugs with weaker inhibitory effects on respiration and circulation, including remimazolam, etomidate, and dexmedetomidine. Studies comparing the clinical efficacy and safety of different anesthesia depths in painless gastrointestinal endoscopy have yielded inconsistent conclusions (Grape et al., 2019). Hou Haijun (Semenas et al., 2020) compared moderate sedation maintained by sequential injection of sufentanil and midazolam with deep sedation maintained by sequential injection of sufentanil and a propofol-etomidate mixture in elderly patients undergoing painless gastroscopy, and concluded that maintaining a certain depth of anesthesia with deep sedation could improve patient comfort and cooperation, significantly increase doctor’s operational satisfaction, reduce heart rate and blood pressure variability, and decrease the incidence of adverse events (Smiley and Moore, 2007; Cao et al., 2021). Wei Xiaozhen et al. (Chen et al., 2020) compared three anesthesia regimens in hospitalized patients with comorbidities undergoing painless gastroscopy: moderate sedation group (midazolam + sufentanil), deep sedation group 1 (propofol + sufentanil), and deep sedation group 2 (propofol + midazolam + sufentanil). They found that combining midazolam and reducing propofol dosage could reduce sedation-related complications. For most hospitalized patients with comorbidities and ASA classification II-III, both moderate and deep sedation are safe and effective for routine upper gastrointestinal endoscopy, but moderate sedation is recommended for patients with unstable circulation (Ozbilgin et al., 2021). Gu Meirong (Kintu et al., 2019) compared conscious analgesia (fentanyl + dicaine mucilage) with deep sedation (propofol) in painless gastroscopy and concluded that conscious sedation anesthesia was more effective, with stable hemodynamic indicators, lower incidence of adverse reactions, and higher clinical safety (Lu et al, 2018), which is consistent with the findings of our study.

In this experiment, surface anesthesia with lidocaine gel combined with intravenous remifentanil injection, based on reducing oropharyngeal sensitivity, effectively inhibited the stress response and cough reflex caused by gastroscopy diagnosis and treatment through the potent analgesic effect of remifentanil, and strongly suppressed the neuroendocrine system stress response (Feng et al., 2021), including biopsy and polypectomy. Specifically, remifentanil acts on μ-opioid receptors in the medial subnucleus of the solitary tract, inhibiting the transmission of pharyngeal reflex signals (Zhou et al., 2022), which is supported by our data showing zero choking incidence in the observation group (vs. 8.2% in the control group). In the neural pathway of the pharyngeal reflex, mechanical stimulation of the tongue root and posterior pharyngeal wall is transmitted via afferent fibers of the trigeminal nerve, glossopharyngeal nerve, and vagus nerve to neurons in the nucleus of the solitary tract and ambiguous nucleus (Tesoro et al., 2021; Kloka et al., 2020), which then project to the palatopharyngeal muscle, gastrointestinal smooth muscle, and abdominal muscles via efferent nerves, causing muscle movements characterized by spasm and uncoordinated vomiting. The nucleus of the solitary tract is the vomiting center, and there are opioid receptors in the medial subnucleus of the nucleus of the solitary tract. A case report (Zhou et al., 2022) demonstrated that after injecting fentanyl and bupivacaine into the subarachnoid space, the pharyngeal reflex disappeared, and it recovered after subsequent administration of naloxone, confirming that opioid drugs inhibit the pharyngeal reflex through opioid receptors, which may be one of the mechanisms.

This study has several limitations. First, it is a single-center study conducted in a coal industry hospital, so the results may not be generalizable to other populations (e.g., urban hospitals or pediatric patients). Second, the follow-up time was limited to 24 h, and long-term outcomes (e.g., delayed nausea) were not evaluated. Third, the study did not adopt a blind design, which may introduce observer bias. Fourth, cost-effectiveness analysis was not performed to compare the economic benefits of the two regimens. Future multi-center, double-blind studies with longer follow-up are needed to validate our findings.

Notably, remifentanil is known to potentially cause chest wall rigidity and postoperative hyperalgesia (Zhou et al., 2022). However, these side effects were not observed in our study, possibly due to the low dosage (0.8–1.2 μg/kg) and short administration duration, which reduces the risk of such adverse reactions.

## 5 Conclusion

Regardless of the depth of sedative anesthesia adopted, the primary goal is to eliminate pain and adverse stimuli caused by diagnosis and treatment. On this basis, it is necessary to weigh the benefits and drawbacks of adverse events and anterograde amnesia brought about by sedation. The mild sedation regimen used in this experiment allows patients to be painless, quiet, and able to cooperate to a limited extent during painless gastroscopy diagnosis and treatment, significantly reducing adverse reactions and events. This regimen is particularly suitable for patients with organ dysfunction due to consumption or those with difficult airways.

## Data Availability

The original contributions presented in the study are included in the article/supplementary material, further inquiries can be directed to the corresponding author.
